# Northern Lancet

**Published:** 1850-05

**Authors:** 


### ARTICLE VIII.

### NORTHERN LANCET.

We have received No II of this new candidate for public favor. It seems to be of mixed character; and aims to cater to the taste of the physician, lawyer, and general reader. We doubt whether the plan is feasible: nevertheless we wish it abundant success.

It is published at Plattsburgh, N. Y. monthly, at $1 per annum, in advance.

